# Supplementary material for: Direct observations of energy transfer from resonant electrons to whistler-mode waves in magnetosheath of Earth
Source: Nat Commun. 2022 Oct 28;13:6259. doi: 10.1038/s41467-022-33604-2 (PMC9616889; doi:10.1038/s41467-022-33604-2)
Supplement: Supplementary file 1 — Supplementary Information [file 41467_2022_33604_MOESM1_ESM.pdf]

Supplementary Information for

Direct observations of energy transfer from resonant electrons to whistler-mode waves in magnetosheath of Earth

N. Kitamura[1,2], T. Amano[2], Y. Omura[3], S. A. Boardsen[4,5], D. J. Gershman[4], Y. Miyoshi[1], M. Kitahara[6], Y. Katoh[6], H. Kojima[3], S. Nakamura[1], M. Shoji[1], Y. Saito[7], S. Yokota[8], B. L. Giles[4], W. R. Paterson[4], C. J. Pollock[9], A. C. Barrie[4,10], D. G. Skeberdis[4,11], S. Kreisler[4,10], O. Le Contel[12], C. T. Russell[13], R. J. Strangeway[13], P.-A. Lindqvist[14], R. E. Ergun[15], R. B. Torbert[16,17], and J. L. Burch[17]

1, Institute for Space-Earth Environmental Research, Nagoya University, Nagoya, Japan.
2, Department of Earth and Planetary Science, Graduate School of Science, the University of Tokyo, Tokyo, Japan.
3, Research Institute for Sustainable Humanosphere, Kyoto University, Uji, Japan.
4, NASA Goddard Space Flight Center, Greenbelt, Maryland, USA.
5, Goddard Planetary Heliophysics Institute, University of Maryland in Baltimore, Maryland, USA.
6, Department of Geophysics, Graduate school of Science, Tohoku University, Sendai, Japan.
7, Institute of Space and Astronautical Science, Japan Aerospace Exploration Agency, Sagamihara, Japan.
8, Department of Earth and Space Science, Graduate School of Science, Osaka University, Toyonaka, Japan.
9, Denali Scientific, Fairbanks, Alaska, USA.
10, Aurora Engineering, Potomac, Maryland, USA.
11, a.i. solutions Inc., Lanham, Maryland, USA.
12, Laboratoire de Physique des Plasmas, CNRS/Sorbonne Université/Université Paris-Saclay/Observatoire de Paris/Ecole Polytechnique Institut Polytechnique de Paris, Paris, France.
13, Department of Earth, Planetary, and Space Science, University of California, Los Angeles, California, USA.
14, Royal Institute of Technology, Stockholm, Sweden.
15, Laboratory for Atmospheric and Space Physics, University of Colorado, Boulder, Colorado, USA.
16, Department of Physics, University of New Hampshire, Durham, New Hampshire, USA.
17, Southwest Research Institute, San Antonio, Texas, USA.
Corresponding author: Naritoshi Kitamura (kitamura@isee.nagoya-u.ac.jp)

Supplementary Information includes:
Supplementary Figs. 1–9

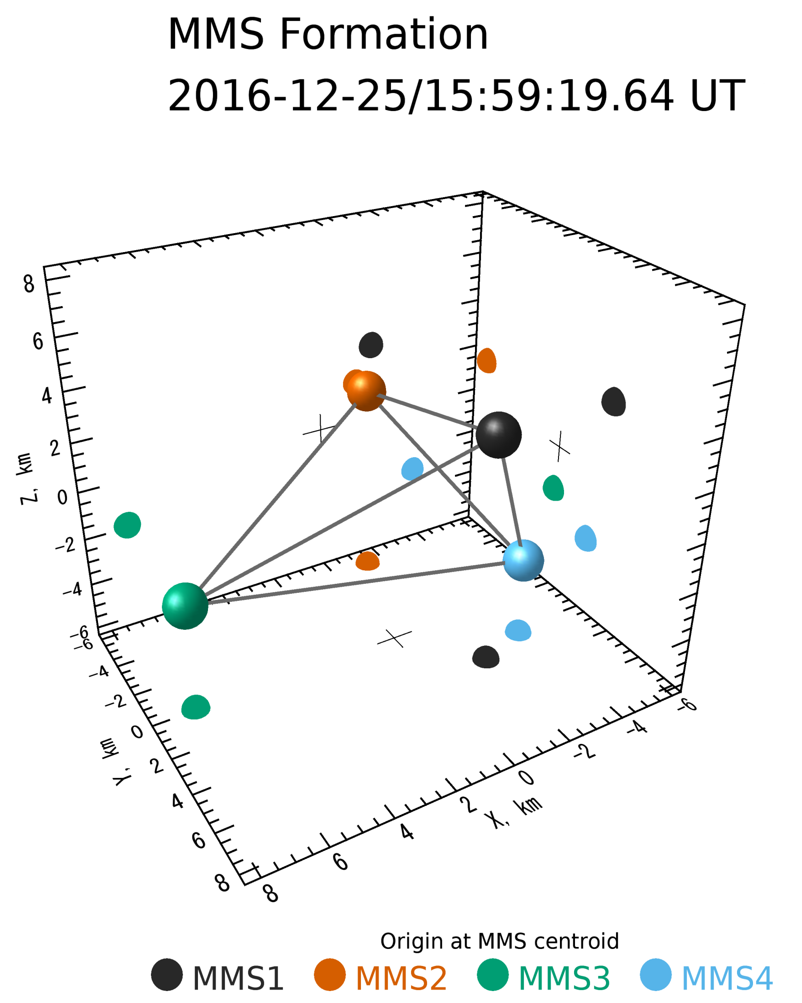

Supplementary Fig. 1 | Formation of MMS spacecraft in the field-aligned coordinate system. MMS1 and MMS4 are the closest pair in the direction perpendicular to the background magnetic field ($\mathbf{B}_0$). The distance perpendicular to $\mathbf{B}_0$ (about 2 km) was comparable to the gyro-radius (about 1.43 km) of the strongly nongyrotropic electrons (Background magnetic field intensity: 33.1 nT, Energy: about 500 eV, Pitch angle: about 141°). The distance along $\mathbf{B}_0$ was about 6 km, which was about 2.5 times smaller than the wavelength of the whistler-mode wave (about 15 km). Even the maximum separation (about 11 km) was much smaller than the gyro-radius of ions (about 116 km) with energy of 700 eV (ion temperature perpendicular to $\mathbf{B}_0$).

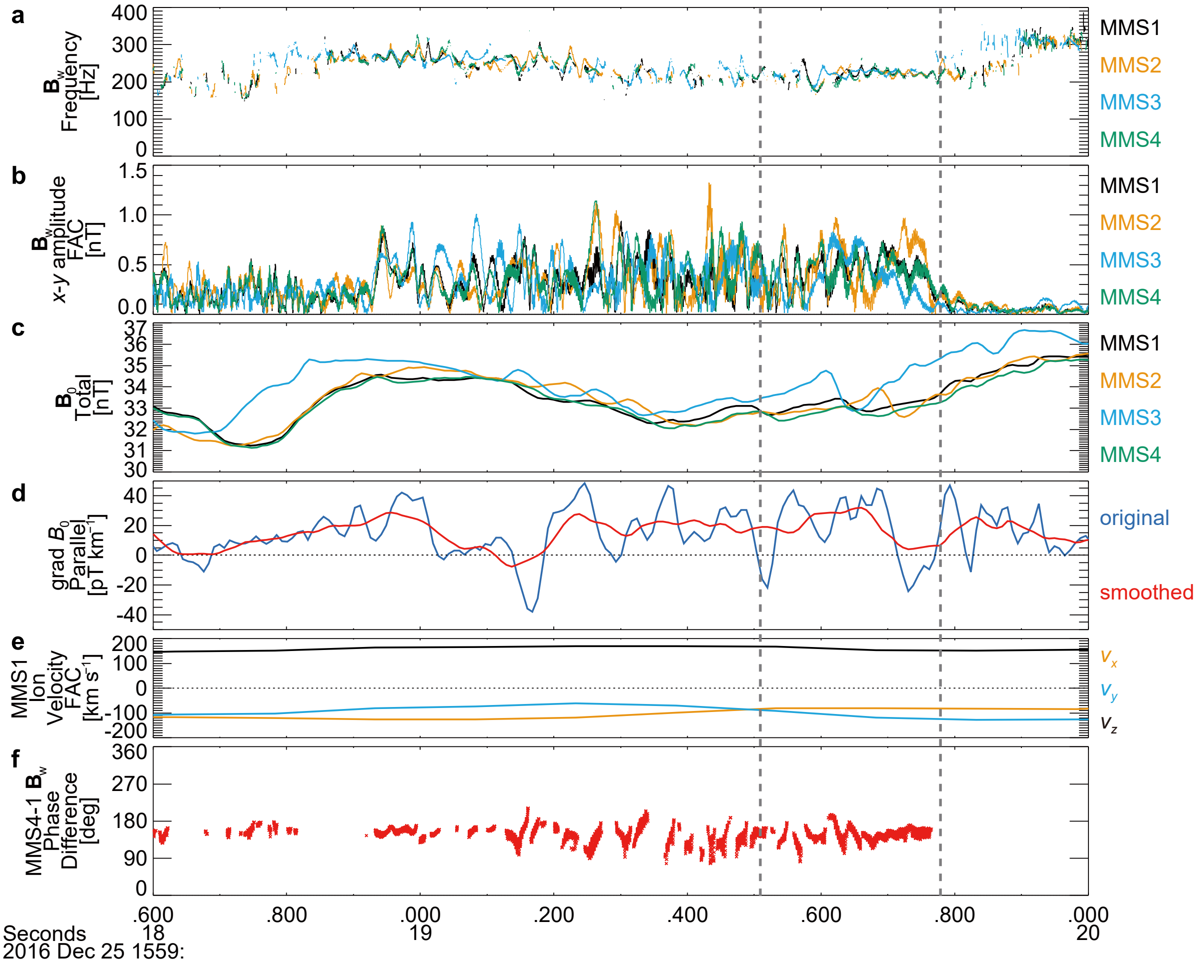

Supplementary Fig. 2 | Details of the whistler-mode wave and gradient of the background magnetic field intensity (Event 1). a. Wave frequency calculated using the waveform of $\mathbf{B}_\mathrm{w}$ (see Methods, subsection Calculation of wave frequency). **b.** Amplitude of $\mathbf{B}_\mathrm{w}$ in the plane perpendicular to the background magnetic field (x-y plane in the field-aligned coordinate (FAC)). **c.** the background magnetic field intensity (B_0). **d.** Gradient of B_0 along the background magnetic field ($\mathbf{B}_0$). Original B_0 and 0.1-s moving-average B_0 were used for blue and red curves, respectively. **e.** Ion bulk velocity in FAC (MMS1). **f.** Phase differences from MMS1 to MMS4 (closest pair in the direction perpendicular to $\mathbf{B}_0$ (see Supplementary Fig. 1)). Vertical grey dashed lines indicate the interval used for Fig. 3.

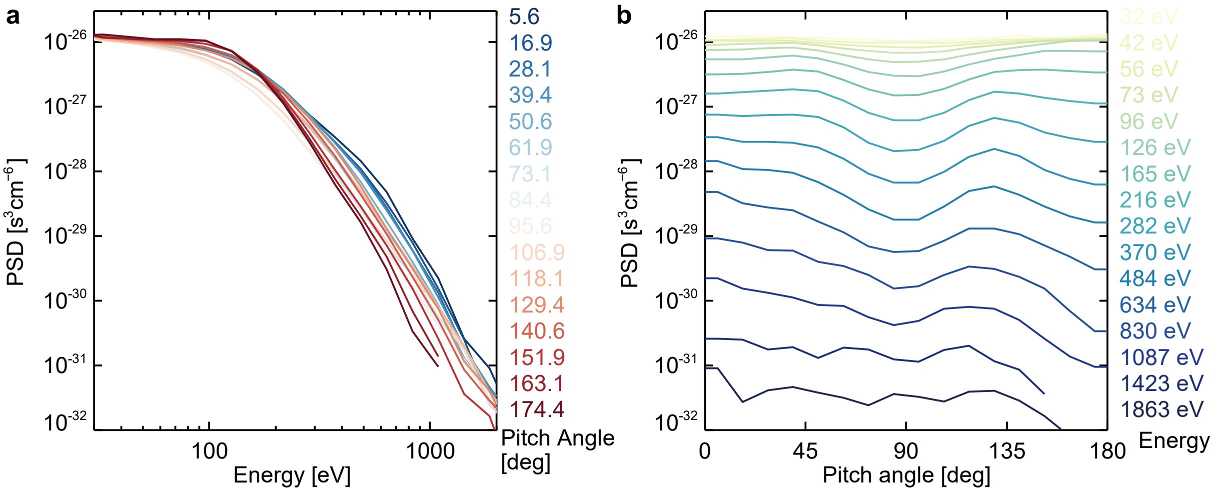

Supplementary Fig. 3 | Details of electron velocity distribution function. a. Energy spectra of electron phase space density (PSD) at various pitch angle (PA) bins. **b.** PA spectra of electron PSD at various energy bins. Electron data from 9 temporal bins (270 ms) around 15:59:19.644 UT (Fig. 3) from MMS1 were used. A gradient of PSD adequate for the initial linear growth of whistler-mode waves (increase toward PA of 90°) is found only at PA larger than about 130° and the energy higher than about 200 eV.

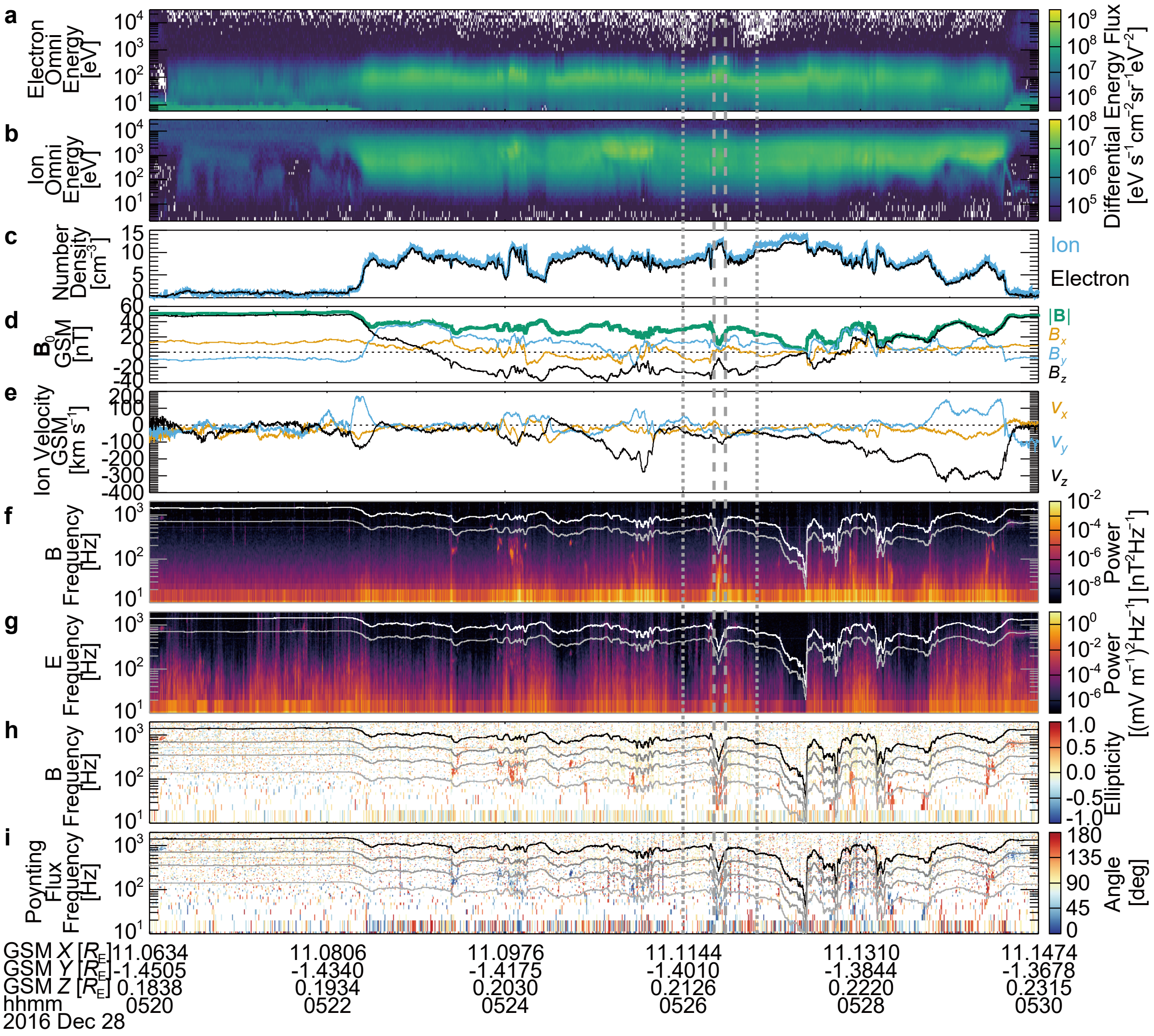

Supplementary Fig. 4 | Long overview of MMS1 burst data (Event 2). The format is the same as that of Fig. 1**.** Vertical gray dotted lines indicate the interval shown in Supplementary Fig. 5. Vertical grey dashed lines indicate the 8-s interval used for the detailed analysis.

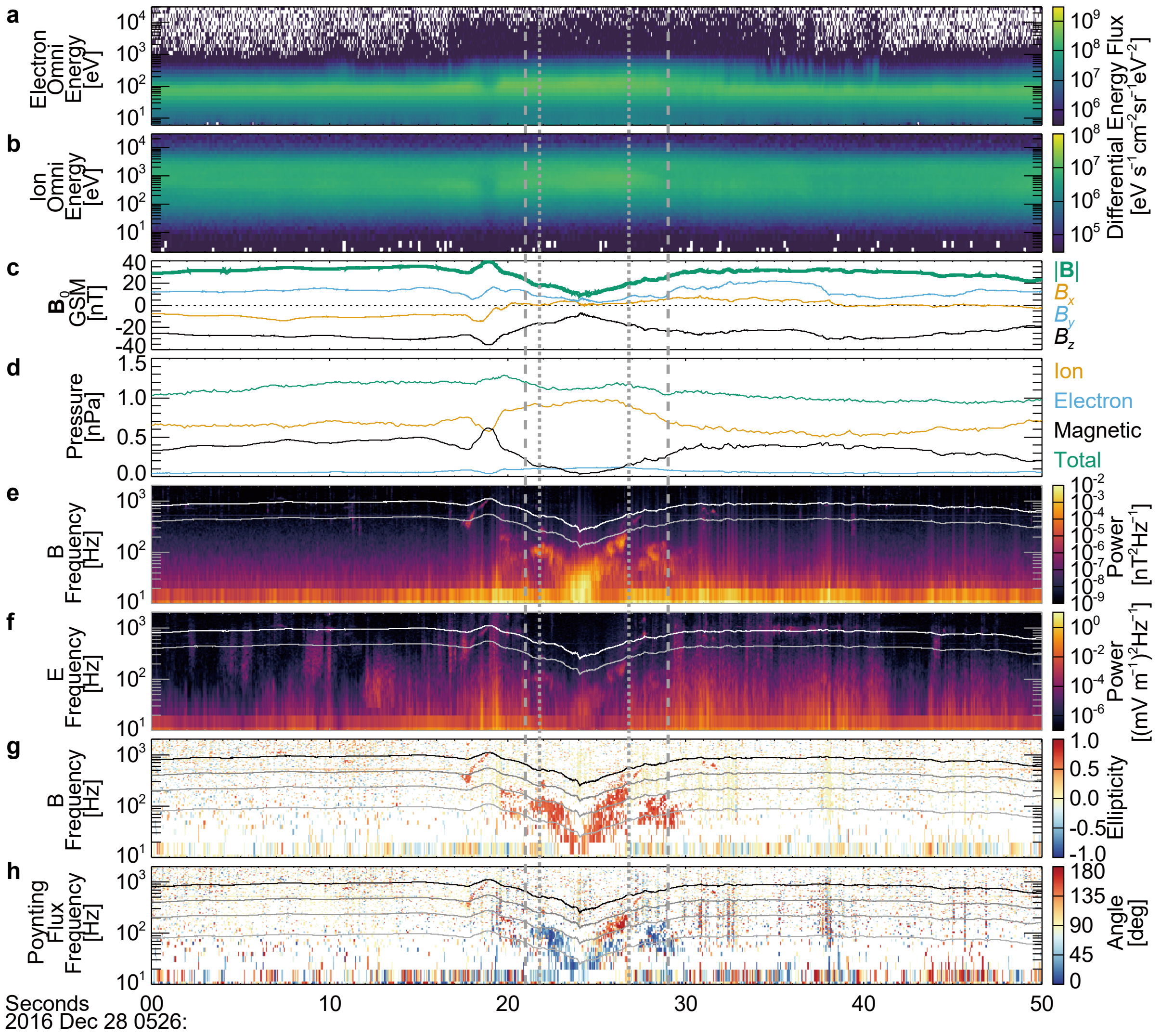

Supplementary Fig. 5 | Short overview of MMS1 burst data (Event 2). a. Omni-directional energy spectrum of electrons (photoelectrons were subtracted). **b.** Omni-directional energy spectrum of ions. **c.** the background magnetic field ($\mathbf{B}_0$) in the geocentric solar magnetic (GSM) coordinates. **d.** Ion, electron, magnetic, and total pressures. In the trough of magnetic field intensity, the decrease in magnetic pressure was compensated with an increase in plasma (mainly ion) pressure. The format of **e–h**, which indicate property of the wave, is the same as that of Fig. 1f–i**.** Enhancements of electromagnetic right-hand polarized (positive ellipticity) waves corresponds to whistler-mode waves. Vertical grey dashed lines indicate the 8-s interval used for the detailed analysis. Vertical gray dotted lines indicate the interval shown in Fig. 6.

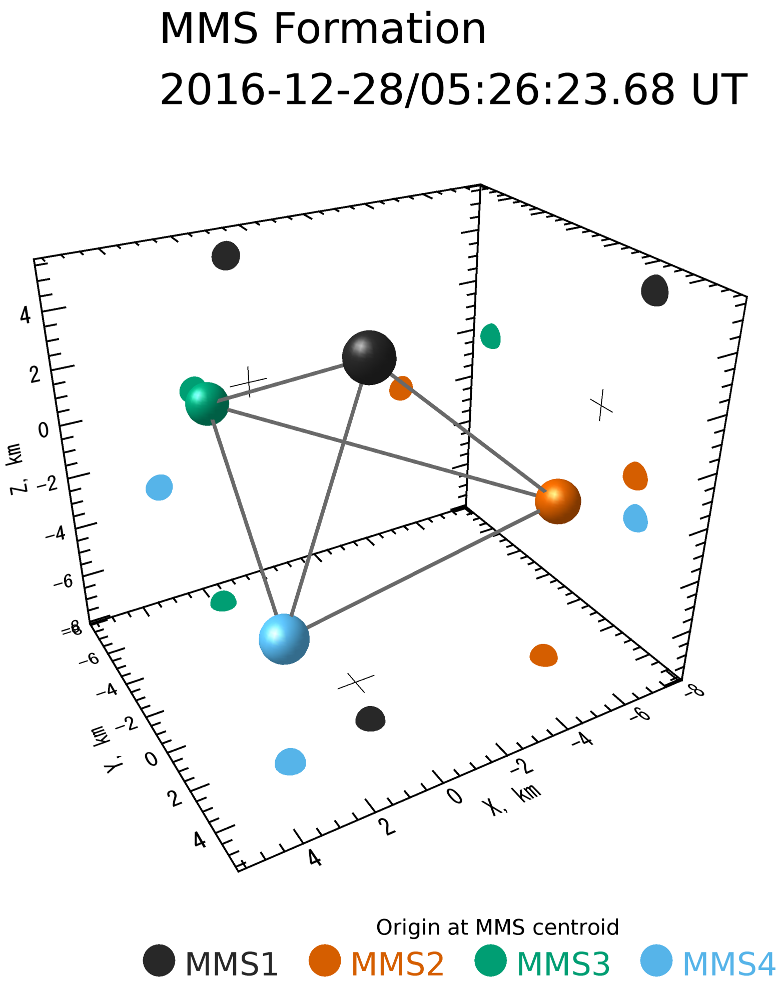

Supplementary Fig. 6 | Formation of MMS spacecraft in the field-aligned coordinate system. MMS1 and MMS4 are the closest pair in the direction perpendicular to the background magnetic field ($\mathbf{B}_0$). The distance perpendicular to $\mathbf{B}_0$ (about 3 km) was smaller than the gyro-radius (about 4.1 km) of the strongly nongyrotropic electrons (Background magnetic field intensity: 11.6 nT, Energy: about 500 eV, Pitch angle: about 141°). The distance along $\mathbf{B}_0$ was about 8 km, which was about 4.3 times smaller than the wavelength of the whistler-mode wave (about 34 km). The maximum separation (about 12 km) was much smaller than the gyro-radius of ions (about 175 km) with energy of 500 eV (ion temperature perpendicular to $\mathbf{B}_0$).

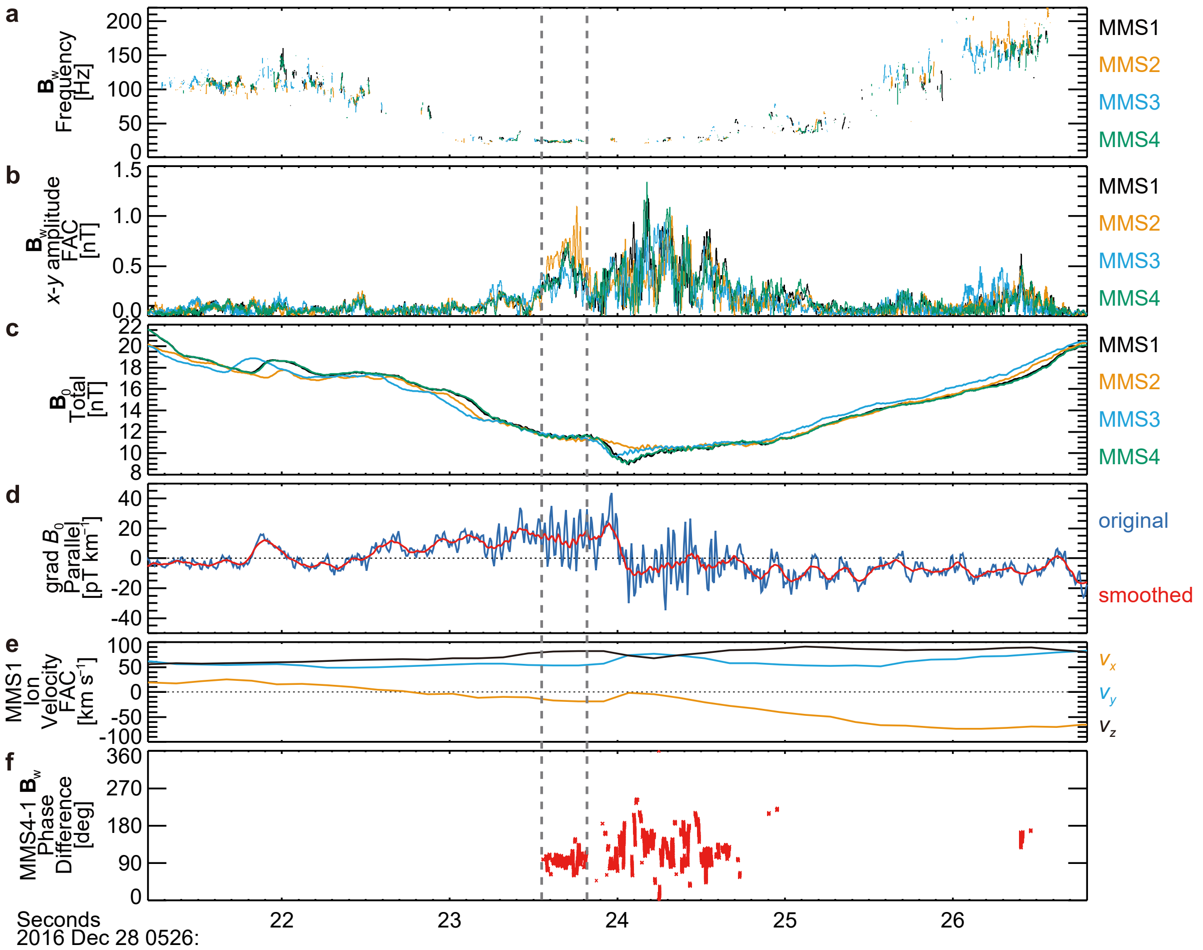

Supplementary Fig. 7 | Details of the whistler-mode wave and gradient of B_0 (Event 2). The format is same as that for Supplementary Fig. 2. MMS1 and MMS4 was the closest pair in the direction perpendicular to the background magnetic field ($\mathbf{B}_0$) (see Supplementary Fig. 6). Vertical grey dashed lines indicate the interval used for Supplementary Fig. 8, 9.

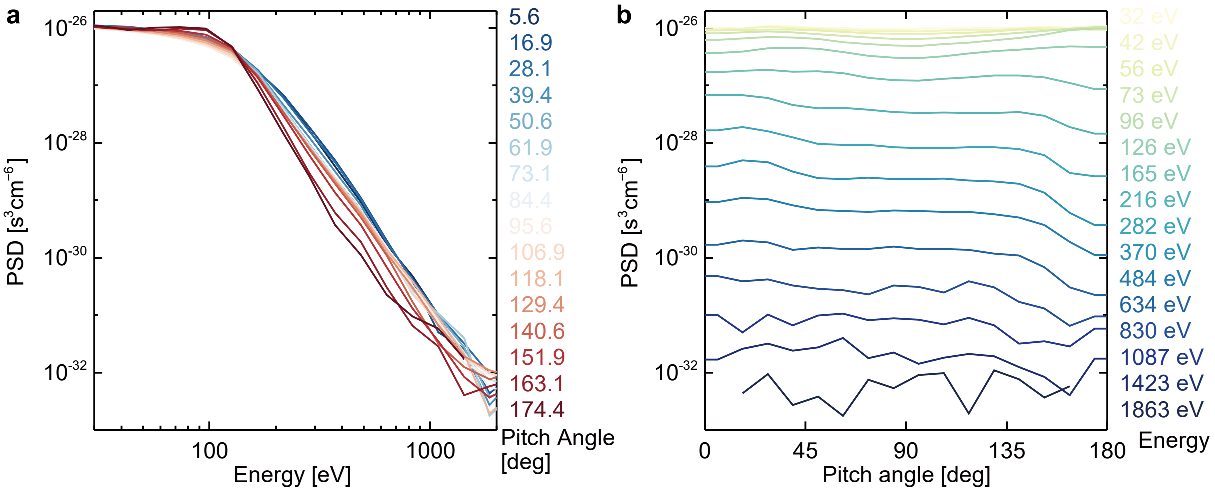

Supplementary Fig. 8 | Details of electron velocity distribution function. The format is same as that for Supplementary Fig. 3. Electron data from 9 temporal bins (270 ms) around 05:26:23.684 UT (Fig. 6) from MMS1 were used. A gradient of phase space density (PSD) adequate for the initial linear growth of whistler-mode waves (increase toward pitch angle (PA) of 90°) is found only at PA larger than about 140° and the energy higher than about 100 eV.

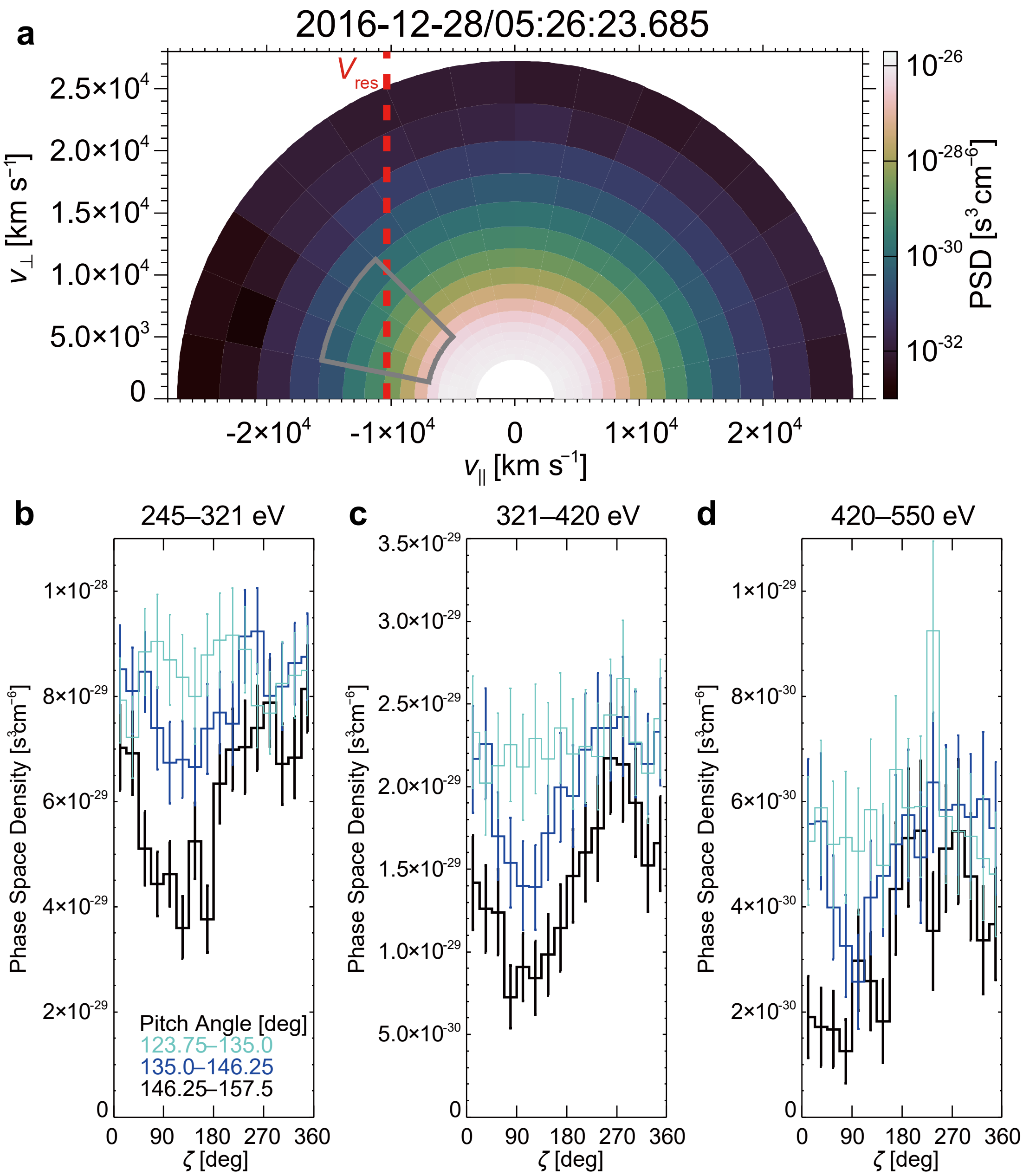

Supplementary Fig. 9 | Electron velocity distribution function (Event 2). a. Gyro-averaged electron velocity distribution function (28.3–2,112 eV) with the resonance velocity (V_{res}) as a red dashed line. **b–d.** histograms of electron PSD in ζ direction at 3 energy bins. The nongyrotropy exceeded 2σ error bars (see Methods, subsection Electron and ion measurements by FPI) around V_{res}, while the electrons did not exhibit clear nongyrotropy around the pitch angle of about 135°. Electron data from 9 temporal bins (270 ms) around 05:26:23.684 UT (Fig. 6) from each spacecraft were used (see Methods, subsection Electron and ion measurements by FPI). The fan-shaped area surrounded by a gray curve indicate the pitch angle and energy ranges for the calculation of the resonant current ($\mathbf{J}_{\mathrm{res}}$) for Event 2.
